# Neighbourhood socio‐economic environment predicts adiposity and obesity risk in children under two

**DOI:** 10.1111/ijpo.12964

**Published:** 2022-08-17

**Authors:** Shannon C. Conrey, Allison R. Burrell, Cole Brokamp, Rachel M. Burke, Sarah C. Couch, Liang Niu, Claire P. Mattison, Alexandra Piasecki, Daniel C. Payne, Mary A. Staat, Ardythe L. Morrow

**Affiliations:** ^1^ Department of Environmental and Public Health Sciences University of Cincinnati College of Medicine Cincinnati Ohio USA; ^2^ Department of Infectious Disease Cincinnati Children's Hospital Medical Center Cincinnati Ohio USA; ^3^ Department of Biostatistics and Epidemiology Cincinnati Children's Hospital Medical Center Cincinnati Ohio USA; ^4^ Division of Viral Diseases Centers for Disease Control and Prevention Atlanta Georgia USA; ^5^ Department of Rehabilitation, Exercise and Nutrition Sciences University of Cincinnati College of Allied Health Sciences Cincinnati Ohio USA; ^6^ Cherokee Nation Assurance Arlington Virginia USA; ^7^ Division of Foodborne, Waterborne, and Environmental Diseases Centers for Disease Control and Prevention Atlanta Georgia USA

**Keywords:** breastfeeding, child obesity, health disparities, social determinants of health, socio‐economic environment

## Abstract

**Background:**

Neighbourhood socio‐economic environment (SEE) is associated with obesity in older children and adults, but little is known about this relationship in younger children. Breastfeeding is an important preventative of adiposity in childhood, but its relationship with neighbourhood SEE is unknown.

**Aims:**

We assessed differences in adiposity and obesity in children before age two by neighbourhood SEE, controlling for family socio‐demographics and breastfeeding duration.

**Materials and Methods:**

Family socio‐demographics, child body mass index *z* scores (BMI*z*), and breastfeeding duration were collected at periodic study visits from participants in PREVAIL (*n* = 245), a birth cohort in Cincinnati, OH. Addresses were assigned a Deprivation Index score, a validated measure of SEE, and dichotomized into highest SEE (least deprived quartile of scores) and not highest SEE (remaining quartiles). Longitudinal and Poisson models assessed differences in BMI*z* by SEE over the second year of life and obesity risk at age two, respectively (highest SEE, *reference*), while attenuation of obesity risk by breastfeeding duration was tested in mediation models.

**Results:**

Residing outside of the highest SEE neighbourhoods was associated with an increased BMI*z* of 0.04 (95%CI 0.02, 0.06) per month of life and increased obesity risk at age two (aRR: 3.7, 95%CI 1.2, 16.2), controlling for family socio‐demographics. Breastfeeding duration attenuated >9% of the obesity risk attributable to SEE (mediated RR: 3.4, 95%CI 1.1, 14.8).

**Discussion:**

In the PREVAIL Cohort, residing outside of the highest SEE neighbourhoods predicted a significant increase in BMI*z* and obesity risk in children before age two, a relationship that was partially mediated by breastfeeding duration.

**Conclusion:**

Breastfeeding support may play an important role in reducing obesity rates in children in lower SEE neighbourhoods.

## 1 INTRODUCTION

Nearly 14% of American children aged 2–5 have obesity, a rate that has more than doubled in the last 50 years.^1^, ^2^, ^3^ In the United States (U.S.), children from low‐ and middle‐income families are more likely to have obesity than their higher‐income peers, and Black children have higher obesity rates than non‐Hispanic White children in all age groups.^1^, ^3^, ^4^ Reducing childhood obesity is a public health priority, as early obesity is a strong predictor of obesity in adolescence and adulthood and increases the risk of a wide range of metabolic, vascular, and endocrine disorders throughout life.^5^, ^6^, ^7^

Evidence shows that weight status and obesity in school age children, adolescents, and adults are intimately connected to a complex network of social, structural, racial, and economic differences in neighbourhood environments.^8^, ^9^, ^10^, ^11^, ^12^ While this relationship in children under two is understudied, socio‐demographic measures associated with differences in child obesity risk, namely, race and family income, are correlated with one's residential neighbourhood^13^, ^14^ and factors that influence parent and sibling weight status also affect the weight status of younger children in the household.^15^, ^16^

Duration of breastfeeding, particularly exclusive breastfeeding, has been associated with reduced risk of obesity in childhood.^17^, ^18^ Disparities in breastfeeding initiation, duration, and exclusivity have been found by maternal race, income, and education level^19^, ^20^ and may, at least partially, explain differences in obesity rates by these socio‐demographic factors.

While disparities in weight status have been found by race and income in older children and adults and these same factors predict both breastfeeding behaviours and neighbourhood socio‐economic environment (SEE), no study in the U.S. has examined these relationships in early childhood as predictors of adiposity and obesity risk. This project seeks to address this knowledge gap using a validated measure of neighbourhood SEE and family‐level factors as predictors of adiposity and obesity, including how breastfeeding behaviours affect these relationships, in a well‐characterized birth cohort. Identifying neighbourhood SEE as a predictor of early obesity could provide public health practitioners with a simple metric for focusing services and interventions in neighbourhoods with the greatest concentration of risk, potentially reducing health disparities through the lifespan.

## 2 MATERIALS AND METHODS

This project is a secondary analysis using data from the Pediatric Respiratory and Enteric Virus Acquisition and Immunogenesis Longitudinal Cohort (PREVAIL), a prospective two‐year birth cohort study in Cincinnati, OH funded by the U.S. Centers for Disease Control and Prevention (CDC). PREVAIL was approved by the Institutional Review Boards at the CDC and Cincinnati Children's Hospital Medical Center. Recruitment, enrollment, and study methods have previously been described^21^; methods relevant to this work are described here. Women 18 years of age and older were provisionally enrolled in the third trimester of pregnancy, with final eligibility determined at a two‐week post‐partum home visit. Inclusion criteria for study mothers included delivery of a healthy, live‐born, singleton infant at one of two urban study hospitals. Exclusion criteria included living more than 20 miles from the birth hospital, illicit drug use during pregnancy, major congenital anomaly in the infant, gestational age under 35 weeks, or HIV infection. Subject enrollment began in April of 2017, and data collection was completed in October 2020. No additional exclusion or inclusion criteria were applied for this analysis.

For this study, we focused on SEE, defined as aggregate socio‐economic indicators of an environment or neighbourhood, such as average income, education, or house value. Family residential address was reported at the baseline visit and updated, if changed, in REDCap data management software.^22^ All PREVAIL subject addresses were geocoded to the census tract‐level, generally aligned with urban neighbourhoods, and assigned a Deprivation Index score^23^ using DeGAUSS software.^24^

The Deprivation Index is a validated measure of SEE using six American Community Survey^25^ census tract‐level variables: (1) percentage of vacant homes, (2) median home value, and the percentage of adult population who (3) are without a highest school diploma, (4) have used any government social‐services or income support within 12 months, (5) lack health insurance, and (6) meet the federal definition of poverty.^25^ Values within each census tract were combined using a principal components analysis and standardized into a score between 0 and 1, with increasing scores representing increasing neighbourhood deprivation.^23^ Differences in child health‐related outcomes have been found by Deprivation Index score, including all‐cause hospitalization during the first year of life, emergency room use, and asthma incidence.^23^, ^26^, ^27^ This study is novel in comparing early childhood obesity rates using this measure.

Quartiles of Deprivation Index score were calculated from assigned scores at all time‐points. Census tracts were categorized by quartile of score into highest SEE (least deprived quartile), high‐mid SEE and low‐mid SEE (intermediate quartiles), lowest SEE (most deprived quartile) and a binary measure of the least deprived quartile (highest SEE) compared to the remaining cohort (not highest SEE). For all comparisons, the Deprivation Index category that corresponded with the measurement timing was used.

Family‐level subject data were collected in‐person at baseline (third trimester) and at in‐clinic visits (week 6, and months 6, 12, 18, and 24). Maternal race was categorized as White, Black, or other (biracial, Asian, Native American, Pacific Islander, or unknown). Maternal age was calculated based on the mother's age at the time of the child's birth. Maternal education level was dichotomized, with levels corresponding to completion of any post‐secondary education or training (>HS) or less (≤HS). Marital status was defined as married or not, regardless of cohabitation status. Insurance status was classified as public or private. There were a few uninsured participants and those who indicated use of both public and private insurance; these were classified as publicly insured. Family income level was categorized in line with the Federal Poverty Level for a family of four (<$25 000/year), between 100% and 200% of Federal Poverty Level ($25 000–$50 000/year) and above (>$50 000/year).

Body mass index *z* scores (BMI*z*) from each study visit were calculated using the parameters provided by the CDC National Center for Health Statistics and the LMS method outlined by Flegal et al.,^28^, ^29^ specific to child sex and age in months at the time of measurement. Imputation for erroneous entries (<0.5% of all entries) was performed by averaging the BMI*z* score from the two proximal visits. Sensitivity analysis found no significant differences in outcomes after removing these imputations. Obesity was defined as a BMI*z* score at or above the 95th percentile (BMI*z* ≥ 1.65) at age two per CDC obesity criteria.^1^ For international comparison purposes, obesity rates at age two were also reported as defined by the International Obesity Task Force (IOTF) and the World Health Organization (WHO)^30^, ^31^; all analysis was performed using CDC criteria.

Breastfeeding duration and exclusivity (dates of cessation and formula introduction, respectively) were self‐reported by the mother on quarterly study questionnaires. Duration of any breastfeeding was calculated in weeks from birth to the maternal reported date of breastfeeding cessation, censored at age two. Duration of exclusive breastfeeding was calculated in weeks from birth until the mother's reported date of the first formula introduction, censored at 6 months of age.

### 2.1 Statistical analysis

Power for each outcome, calculated post‐hoc, was based on the smallest sample size at each study visit for any outcome. Our study had at least 80% power to detect an effect size of ≥30% using any of our analytic methods.

To characterize the study population, family‐level socio‐demographics by categorical and binary SEE were compared using Fisher's exact test. Spearman correlations between categories of SEE (ranked 1–4, lowest to highest) and each family‐level characteristic were calculated. Variables with a strong correlation (*r* ≥ 0.70) were considered collinear, and not considered for inclusion in multivariable models. Family‐level variables that met non‐collinearity criteria were used in all subsequent models unless otherwise indicated.

Our analytic approach was designed to identify and track differences in BMI*z* by SEE in early childhood and subsequent risk of obesity at age two. In line with published CDC findings of increased obesity risk for low and middle income compared to high‐income young children,^4^ binary highest vs not highest SEE categories were compared. To test for a possible dose–response effect, models including categorical SEE were also constructed. Child BMI*z* at 6 weeks and 6 months did not meet variance or normality assumptions, so BMIz was evaluated beginning at 12 months of age. Mean BMI*z* was compared using ANOVA at each time‐point, with Tukey adjustments for multiple comparisons. To test individual total effects of SEE and family‐level variables, linear regression models were run, first with categorical and binary SEE and each family‐level variable in univariable analyses, then in multivariable regression models including categorical or binary SEE and all non‐collinear family‐level variables.

To estimate BMIz differences over the second year of life, a generalized estimating equation (GEE) with an exchangeable correlation matrix was constructed using BMI*z* data from the 12, 18, and 24 month study visits. Each variable of SEE and family socio‐demographics was run as a univariable GEE model, then a multivariable GEE model with binary SEE and family‐level predictors was constructed. Based on the time‐point specific analysis, the relationship between SEE and BMI*z* scores appeared to differentiate over time, so an interaction term between the child's age in months and SEE was added to the GEE models.

Obesity prevalence at age two was calculated and compared using the CDC, IOTF,^31^ and WHO^30^ standards. The CDC defines obesity as BMIz score ≥ 95th percentile (BMIz ≥ 1.65), whereas the IOTF (male BMI > 20.09, female BMI > 19.81) is based on BMI cut‐points and WHO definitions are based on a BMI*z* ≥ 3, with BMI*z* calculated using WHO LMS criteria. Comparisons of obesity prevalence as calculated by these methods were made using Fisher's exact test. As this analysis includes an American study population, all assessments of obesity risk were made using CDC standards. Relative risk of obesity at age two was calculated using Poisson regression in univariable and multivariable analysis, using the same variable selection approach previously described.

As lack of breastfeeding, particularly exclusive breastfeeding, has been associated with risk of obesity,^17^, ^18^ mediation analysis was undertaken to examine the effects of lack of breastfeeding, not as a confounder, but as a potential causal pathway of obesity. First, restricted mean survival time (RMST) models were constructed to determine if significant differences were found between binary or categorical SEE and any or exclusive breastfeeding duration in those who completed the 24‐month study visit (model *a*). RMST models provide reliable, interpretable estimates of multivariable survival data by comparing estimated mean time to events calculated using areas under the Kaplan Meier survival curves.^32^ Mediation was tested based on significant model *a* results by constructing a series of Poisson regression models controlling for family‐level predictors: (model *b*) obesity status by the duration of any and exclusive breastfeeding; (model *c*) obesity status by SEE (model *c*’) obesity status by SEE and any or exclusive breastfeeding duration. Results were reported as the resulting relative risks and the percent effect mediated (PEM), calculated as the proportion of the effect of SEE attributable to breastfeeding duration (RR model *c*’/RR model *c*). Full mediation was defined as a change in significance level of the SEE variable with a concurrent significant effect of breastfeeding in model *c*’. Partial mediation was defined as a change in the effect size of SEE with a concurrent significant effect of breastfeeding in model c’.

The final quarter of PREVAIL data collection and follow‐up occurred in spring and summer of 2020, which coincided with the early months of the COVID‐19 pandemic. COVID‐19 restrictions resulted in a temporary closure of the study clinic and caused approximately 9% (16/171) of subjects to complete their 24‐month visit outside of the protocol‐established window (within 12 weeks of the child's second birthday). All measures of BMI*z* were calculated using parameters associated with the child's age in months, and GEE models added child's age in months at the time of measurement as an interaction term. However, as differences associated with SEE appeared to increase over time, sensitivity analysis was performed by excluding this subset of subjects from analysis and re‐running the linear regression models of BMI*z*, logistic models of obesity, and mediation models at age two. As no differences were found after these removals, results are reported using all subjects available at the specific study time‐point.

All comparisons were made with the highest SEE census tracts as the reference group. Results are reported as estimate and 95% confidence interval (95% CI), except where indicated. Analysis was performed using the R Environment for Statistical Computing version 4.1.2, including the geepack and survRM2 packages.^33^, ^34^, ^35^

## 3 RESULTS

All subjects enrolled in PREVAIL (*n* = 245) were eligible for inclusion in this analysis. The cohort was racially and economically diverse (Table 1), with 44% of subjects self‐identifying as Black, 51% as white, and 4% as other; one‐third of subjects reported a family income below $25 000/year, and over half reported an income below $50 000/year.

**TABLE 1:** Study demographics by socio‐economic environment

			All *N* = 245	Highest SEE *n* = 56	High‐Mid *n* = 70	Low‐Mid *n* = 56	Lowest SEE *n* = 63	*All categories p*	*High SEE vs Not High SEE p*
Census Tract SEE	Deprivation index score	Median Range	0.392 0.167–0.851	0.256 0.167–0.302	0.360 0.304–0.392	0.456 0.392–0.541	0.677 0.555–0.851	‐	‐
Family‐level factors	Maternal age	mean ± SD	29.8 ± 5.2	32.4 ± 4.5	30.2 ± 4.9	28.9 ± 5.2	27.8 ± 5.3	<0.001	<0.001
	Race	Black	107 (44%)	7 (12%)	17 (24%)	29 (52%)	54 (86%)	<0.001	<0.001
		White	126 (51%)	48 (86%)	47 (67%)	25 (45%)	6 (10%)		
		Other	12 (5%)	1 (2%)	6 (9%)	2 (3%)	3 (4%)		
	Married	Yes	118 (48%)	45 (80%)	44 (63%)	19 (34%)	10 (16%)	<0.001	<0.001
	Education	≤HS	115 (47%)	8 (14%)	27 (39%)	29 (52%)	51 (81%)	<0.001	<0.001
	Annual family income	<$25 000	78 (32%)	5 (9%)	13 (19%)	20 (36%)	40 (63%)	<0.001	<0.001
		$25–50 000	49 (20%)	4 (7%)	16 (23%)	17 (30%)	12 (19%)		
		>$50 000	118 (48%)	47 (84%)	41 (59%)	19 (34%)	11 (17%)		

*Notes* : Study demographics at baseline for the Pediatric Respiratory and Enteric Virus Acquisition and Immunogenesis Longitudinal Cohort (PREVAIL) Cohort by category of socio‐economic environment (SEE). SEE was determined by quartile of Deprivation Index score, a validated measure of census tract‐level SEE. Socio‐demographics were self‐reported by the mother at a pre‐natal baseline visit. Maternal age was calculated at the time of the child's birth. Race was classified as Black, White, or other (biracial, Asian, Pacific Islander or Alaskan native, Native American), insurance was classified as public or private, with subjects reporting no insurance or use of both public and private insurance classified as public. Education was dichotomized to the completion of at least two years of post‐secondary education (>HS) or not (≤HS). Annual family income was categorized in line with the Federal Poverty Level for a family of four (<$25 000), between 100%–200% of Federal Poverty Level ($25–50 000), or above (>$50 000).

Correlation coefficients between category of SEE and family‐level predictors were significant but only weakly to moderately correlated, with maternal age the weakest (*r* = 0.22, *p* < 0.001) and Black race the strongest (*r* = −0.32, *p* < 0.001) correlation. However, use of public insurance was strongly correlated to lower income (*r* = 0.75, *p* < 0.001) and unmarried status (*r* = 0.71, *p* < 0.001), so was excluded from all multivariable models. No other family‐level predictor was strongly correlated (*r* ≥ 0.70) to either SEE or other family‐level variables, so all multivariable models were adjusted by the covariates race, maternal education, family income, maternal age, and marital status (family‐level predictors) unless otherwise indicated.

All family‐level predictors differed significantly by Deprivation Index category (Table 1). Across categories, lower SEE was consistently associated with decreasing maternal age and increasing proportions of Black race, being unmarried, use of public insurance, lower academic achievement, and family incomes below $25 000/year. These differences remained when comparing only the highest to not highest SEE census tracts. For example, only 12.5% of the highest SEE census tracts described themselves as Black, whereas 52.9% of the remaining cohort was Black, with the highest proportion of Black residents found in the lowest SEE census tracts (85.7%).

**FIGURE 1:**
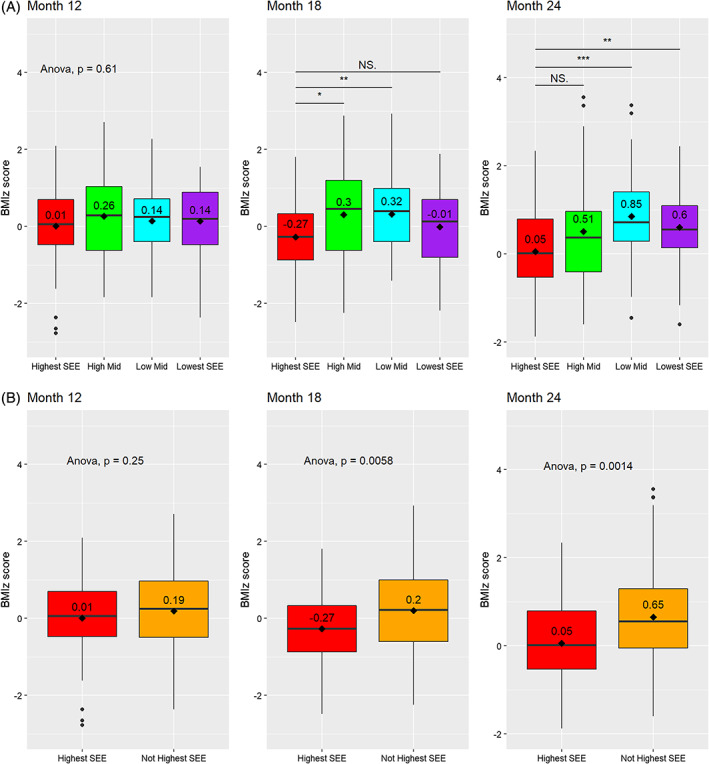
Differences in body mass index *z* scores (BMI*z*) by census tract socio‐economic environment. BMI*z* in children 12, 18, and 24 months of age was calculated using data from the Pediatric Respiratory and Enteric Viral Acquisition and Immunogenesis Cohort (PREVAIL), a CDC‐funded birth cohort in Cincinnati, OH. Neighbourhood socio‐economic environment (SEE) was quantified using a validated measure of census tract material deprivation, the Deprivation Index. Quartiles of score were calculated and SEE was categorized as highest SEE (least deprived census tracts), high mid and low mid (intermediate levels of deprivation) and lowest SEE (most deprived census tracts). Comparisons were made among all categories (A) and by binary highest SEE *vs* all others (not highest SEE) (B). Comparisons of mean BMI*z* were made using ANOVA, with multiple comparisons adjusted using Tukey corrections.*p < 0.05, **p < 0.01, ***p < 0.001

No measure of SEE was significantly associated with BMI*z* at 12 months of age (Figure 1). However, at 18 months mean BMI*z* for children outside of the highest SEE tracts was 0.47 (95% CI: 0.17, 0.78) points higher than those in the highest SEE tracts and 0.60 (95% CI: 0.26, 0.94) points higher at 24 months. Pairwise comparisons revealed that the differences were between the highest SEE and the two intermediate categories, with no significant difference between highest and lowest SEE or lowest and either of the intermediate SEE census tract categories at 18 or 24 months. No other ecological or family‐level predictor was associated with significant differences in mean BMI*z* at any time‐point (data not shown).

The low number of participants who identified as ‘other’ race caused a failure in model convergence in several multivariable regression models, thus; race was dichotomized to Black and Not Black for all modelling purposes. In the time‐point specific univariable regression models (Table 2), residence outside of highest SEE census tracts was associated with significantly increased BMI*z* beginning at 18 months. This relationship was particularly strong at 24 months, with residence in any census tract outside of the highest SEE predicting a significant increase in BMI*z* of 0.60 points (95%CI 0.24, 0.96). There were no significant differences among intermediate and lowest SEE census tracts, and the significant family‐level predictors in univariable analysis, Black race and income < $25 000/year, were associated with a decreased BMI*z* only at 12 months.

**TABLE 2:** Child body mass index *z* scores (BMI*z*) by socio‐economic environment and family‐level factors in the PREVAIL Cohort

Study visit			Month 12		Month 18		Month 24		Months 12–24^a^	
*N*			203		195		171		208 subjects 569 observations	
Estimate			β	*95% CI*	β	*95% CI*	β	*95% CI*	β	*95% CI*
Univariable models	SEE (categorical)	Highest SEE	*reference*		*reference*		*reference*		*reference*	
		High Mid	0.251	−0.116, 0.619	**0.575**	**0.160, 0.989**	**0.452**	**0.006, 0.898**	**0.029**	**0.005, 0.054**
		Low Mid	0.134	−0.266, 0.533	**0.593**	**0.163, 1.022**	**0.800**	**0.342, 1.257**	**0.053**	**0.024, 0.082**
		Lowest SEE	0.127	−0.268, 0.521	0.264	−0.159, 0.688	**0.550**	**0.078, 1.022**	**0.029**	**0.000, 0.059**
	SEE (binary)	Not Highest SEE	0.179	−0.130, 0.487	**0.478**	**0.140, 0.817**	**0.597**	**0.235, 0.960**	**0.037**	**0.016, 0.058**
	Race	Black	**−0.300**	**−0.577, −0.023**	−0.09	−0.404, 0.224	0.197	−0.149, 0.544	−0.149	−0.420, 0.121
	Maternal Age	Years	0.003	−0.025, 0.030	0.007	−0.024, 0.038	0.002	−0.034, 0.038	0.006	−0.020, 0.033
	Married	No	−0.100	−0.377, 0.176	0.081	−0.229, 0.392	0.244	−0.099, 0.588	0.025	−0.243, 0.293
	Income	$25–50 000/year	**−0.361**	**−0.678, −0.043**	−0.004	−0.361, 0.352	0.081	−0.321, 0.483	−0.132	−0.425, 0.160
		<$25 000/year	0.064	−0.301, 0.428	0.235	−0.186, 0.655	0.419	−0.035, 0.873	0.241	−0.146, 0.629
	Education	≤High School	−0.062	−0.341, 0.216	0.217	−0.095, 0.530	0.128	−0.222, 0.478	0.053	−0.217, 0.323
Multivariable model	SEE	Not highest SEE	0.333	−0.01, 0.676	**0.555**	**0.174, 0.936**	**0.611**	**0.200, 1.022**	−0.128	−0.607, 0.352
	Child Age	Month of age	–	–	–	–	–	–	0.001	−0.017, 0.018
	Interaction	SEE × child age (months)	–	–	–	–	–	–	**0.037**	**0.016, 0.059**
	Race	Black	−0.366	−0.744, 0.012	−0.401	−0.823, 0.021	0.020	−0.469, 0.508	−0.367	−0.735, 0.001
	Maternal Age	Years	0.004	−0.028, 0.035	0.025	−0.010, 0.059	0.015	−0.025, 0.054	0.015	−0.014, 0.044
	Married	No	0.103	−0.310, 0.516	0.038	−0.438, 0.514	0.197	−0.311, 0.704	0.098	−0.292, 0.489
	Income	$25–50 000/year	−0.498	−1.010, 0.014	−0.274	−0.842, 0.294	−0.217	−0.876, 0.443	−0.275	−0.698, 0.149
		<$25 000/year	−0.060	−0.524, 0.405	−0.003	−0.531, 0.525	0.120	−0.480, 0.721	0.087	−0.340, 0.515
	Education	≤High School	0.255	−0.187, 0.697	**0.497**	**0.005, 0.989**	−0.068	−0.612, 0.477	0.235	−0.182, 0.651

*Notes*: Child weight and length were measured at each study visit in the Pediatric Respiratory and Enteric Viral Acquisition and Immunogenesis (PREVAIL) Cohort. BMI*z* was calculated using parameters supplied by the U.S. Centers for Disease Control and Prevention. Linear regression models and generalized estimating equations (GEE) were used to examine differences in BMI*z* by measures of neighbourhood socio‐economic environment (SEE) and family‐level socio‐demographics. Neighbourhood socio‐economic environment (SEE) was quantified using a validated measure of census tract material deprivation, the Deprivation Index. Quartiles of score were assigned and SEE was categorized as Highest SEE (least deprived census tracts), high mid and low mid (intermediate levels of deprivation) and lowest SEE (most deprived census tracts). Comparisons were made among all categories and by binary highest SEE vs all others (not highest SEE). Race was dichotomized to Black or not Black (*reference*), maternal age was calculated in years at the time of the child's birth; family income was categorized as <$25 000/year, between $25–$50 000/year, and above $50 000/year (*reference*); maternal education was dichotomized to completion of any post‐secondary education (>HS, *reference*) or not (≤HS). Models were run first as univariable models with the outcome of BMI*z* and each individual predictor, then as a multivariable model including binary SEE and all family‐level predictors. Results are reported as the regression coefficient (β) and the 95% confidence interval (95%CI). Bolded results are statistically significant.

^a^ The estimates for binary and stratified SEE in the univariable GEE model reflect the interaction term with child age in months.

Due to the similarity in estimates for all SEE categories outside of highest SEE, the multivariable regression model was constructed using binary highest SEE vs not highest SEE (Table 2). At month 18, when controlling for family‐level predictors, residing outside of highest SEE and maternal education ≤ high school were associated with an increased BMI*z*, while only the effect of SEE remained significant at 24 months. No other family‐level predictor was significantly associated with BMI*z* in multivariable modelling at any time‐point.

A total of 208 children completed at least one study visit at 12, 18, or 24 months of age, providing 569 observations for GEE models (Table 2). Residing outside of the highest SEE neighbourhoods predicted a significant increase in BMI*z* of approximately 0.04 points for each month in child age in both univariable and multivariable models, translating to a difference of 0.44–0.88 points in BMI*z* for these children from 12–24 months of age. No family‐level predictor was significantly associated with differences in BMIz when generalized from 12–24 months of age.

Internationally, standards for obesity differ from the CDC standards used in the U.S. Using the CDC obesity standard, prevalence of obesity in PREVAIL children at age two was 15.8% (27/171), significantly higher than the prevalence based on IOTF (10/171, 5.9%, *p* < 0.001) and WHO (4/171, 2.3%, *p* < 0.001) definitions. Using only the CDC definitions, we found significant differences when comparing the highest to not highest SEE census tracts (Table 3). Only 5.8% (3/51) of the children in highest SEE tracts had a BMI*z* ≥1.65, whereas 20.0% (24/120) of the remaining cohort had obesity. In univariable Poisson regression, residing outside of the highest SEE census tracts was associated with a 3.4‐fold increase in risk of obesity compared to the highest SEE census tracts. When controlling for family‐level predictors, the risk for obesity in the not highest SEE tracts was 3.7‐fold that of those in the highest SEE tracts. There were no significant differences in obesity risk associated with family‐level predictors in either the univariable or the multivariable obesity models.

**TABLE 3:** Obesity prevalence and risk at age two by measures of socio‐economic environment in the PREVAIL Cohort

			Prevalence	Univariable Poisson models		Multivariable Poisson model	
Variable	Level	N	*n* (%)	RR	95%CI	RR	95%CI
	All subjects	171	27 (15.8%)	–	–	–	–
SEE (stratified)	Highest SEE	51	3 (5.8%)	*ref*		*ref*	
	High Mid	44	9 (20.5%)	**3.477**	**1.038, 15.67**	–	–
	Low Mid	40	8 (20.0%)	3.400	0.983, 15.52	–	–
	Lowest SEE	36	7 (19.4%)	3.306	0.919, 15.33	–	–
SEE (binary)	Not Highest SEE	120	24 (20.0%)	**3.400**	**1.189, 14.31**	**3.722**	**1.209, 16.231**
Race	White	101	15 (15.0%)	*ref*		*ref*	
	Black	70	12 (17.1%)	1.154	0.53, 2.462	0.831	0.295, 2.471
Maternal age	Years			1.036	0.957, 1.123	1.056	0.969, 1.147
Marital status	Married	98	13 (13.3%)	*ref*		*ref*	
	Unmarried	73	14 (19.2%)	1.446	0.676, 3.115	1.654	0.536, 5.297
Income	>$50 000/year	94	13 (13.8%)	*ref*		*ref*	
	<$25 000/year	43	7 (15.6%)	1.125	0.423, 2.746	0.747	0.195, 3.129
	$25–50 000/year	32	7 (21.9%)	1.582	0.594, 3.861	1.017	0.299, 3.412
Maternal Education	>High School	105	16 (15.2%)	*ref*		*ref*	
	≤High School	66	11 (16.7%)	1.094	0.494, 2.336	0.948	0.326, 2.813

*Notes*: Obesity prevalence in the Pediatric Respiratory and Enteric Viral Acquisition and Immunogenesis (PREVAIL) Cohort was determined using U.S. Centers for Disease Control and Prevention criteria (BMIz ≥1.65). Neighbourhood socio‐economic environment (SEE) was quantified using a validated measure of census tract material deprivation, the Deprivation Index. Quartiles of score were assigned and SEE was categorized as Highest SEE (least deprived census tracts), high mid and low mid (intermediate levels of deprivation) and lowest SEE (most deprived census tracts). Comparisons were made among all quartiles and by binary highest SEE vs all others (not highest SEE) as well as by family‐level race, income, and maternal age, marital status, and education. Poisson regression was used to calculate the relative risk (RR) of obesity by each SEE and family‐level variable in univariable models, and then in a combined multivariable model containing the binary measure of SEE and all of the family‐level variables. Reference values are indicated. Bolded results are statistically significant.

Duration of exclusive breastfeeding did not meet criteria for mediation of either categorical or binary SEE, as the difference in duration of exclusive breastfeeding by restricted mean survival time (ΔRMST) was not statistically significant when controlling for family‐level predictors. However, both categorical and binary SEE was significantly associated with differences in any breastfeeding duration; ΔRMST was not significant between highest SEE and high mid, but ΔRMST was significant between highest SEE and low mid (11.3 weeks, 95% CI 8.83, 13.7) and highest and lowest SEE (7.12 weeks, 95% CI 4.63, 9.61) when controlling for family‐level predictors. In the binary SEE model, ΔRMST was 4.13 weeks (95% CI 2.39, 5.88) between highest and not highest SEE. Given the significant relationship between binary SEE and breastfeeding duration, and the significant risk of obesity associated with residing outside of the highest SEE census tracts, mediation was undertaken using the binary SEE measure and duration of any breastfeeding.

**FIGURE 2:**
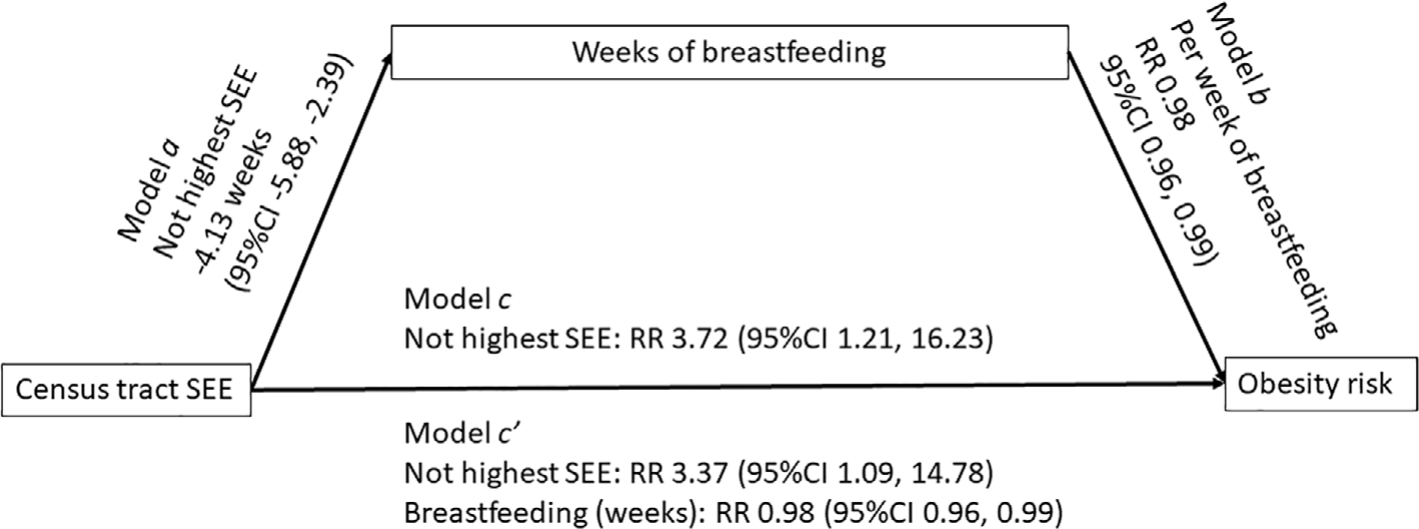
Child weight and length were measured at each study visit in the Pediatric Respiratory and Enteric Viral Acquisition and Immunogenesis (PREVAIL) Cohort and BMI*z* was calculated using parameters supplied by the US Centers for Disease Control and Prevention. Obesity was defined as a BMI*z* ≥ 1.65 at age two. Census tract socio‐economic environment (SEE) was determined based on residing in the least deprived quartile of Deprivation Index score (highest SEE), a validated measure of neighbourhood material deprivation, or not (not highest SEE). Breastfeeding duration was self‐reported by the mother on quarterly study surveys and compared (model *a*) using a restricted mean survival time approach, controlling for family socio‐demographics variables (race, maternal age, marital status, family income, maternal education). Obesity risk by breastfeeding duration (model *b*), SEE (model *c*) and SEE + breastfeeding duration (model *c*’) was compared using Poisson regression while controlling for family socio‐demographics. Significant mediation by breastfeeding duration was defined as a significant effect of breastfeeding with a change in effect size or significance level of SEE in model *c*’

The RR of obesity for not highest compared to highest SEE census tracts was partially mediated by breastfeeding duration (Figure 2). The impact of SEE remained significant but reduced from 3.72 to 3.37 (95% CI 1.09, 14.78), a reduction of risk of 9.4%. Breastfeeding duration in this model remained significantly protective against obesity; with each week of breastfeeding reducing risk of obesity by 2.0% (RR 0.98, 95% CI 0.96, 0.99).

## 4 DISCUSSION

In the PREVAIL Cohort, neighbourhood SEE, as measured by the category of Deprivation Index score, was a significant predictor of increased BMI*z* at 18 and 24 months and increased obesity risk at 24 months. Residence outside of the highest SEE neighbourhoods was associated with an increase of BMI*z* score at 18 and 24 months of age and more than triple the risk of obesity at age two. Examined longitudinally, the differences in BMI*z* by SEE increased incrementally with each month of child age during the second year of life. Differences in BMI*z* and obesity did not have a linear dose–response relationship with level of neighbourhood deprivation. Instead, BMI*z* and obesity were similarly elevated for all groups outside of the highest SEE census tracts. There were few significant associations between family‐level predictors and BMI*z* in univariable and multivariable analyses, and none in any models of obesity risk; controlling for family‐level predictors did not significantly change the association between neighbourhood SEE and adiposity or obesity in any models. While breastfeeding duration did not fully mediate SEE's effect on obesity risk, it does provide a potential pathway for improving obesity rates, decreasing risk of obesity by nearly 2% for every week of breastfeeding continued.

While this study was novel in comparing adiposity and obesity in children under two by neighbourhood deprivation, finding lower obesity in the most affluent group compared to all others is consistent with nationally representative data.^4^ Using the National Health and Nutrition Examination Survey, Ogden et al found no significant difference in obesity rates between children in low (18.9%) and middle income (19.9%) categories, while both significantly differed from the highest‐income group (10.9%).^4^ Although U.S. obesity rates have also been shown to differ by race and education,^1^, ^36^ we did not find differences in obesity by income, race, or maternal education in univariable or multivariable analysis. Indeed, residing outside of highest SEE remained significant when controlling for the family‐level predictors, and adding family‐level data did not significantly change the estimates for SEE.

This may be due to the combined effects of family socio‐demographics and neighbourhood SEE. Neighbourhood deprivation was significantly, but only moderately, correlated with family‐level sociodemographic differences across all categories and our multivariable models met non‐multicollinearity criteria. However, the highest SEE group had significantly lower proportion of low income, Black, and single‐parent families as well as significantly higher education levels compared to low mid and lowest SEE as well as compared to all others, suggesting a high level of socio‐demographic segregation by neighbourhood at either end of the affluence spectrum.

Socio‐demographic segregation in the U.S. has previously been associated with neighbourhood deprivation and health disparities. In their seminal 1993 publication, *American Apartheid*, Massey and Denton quantified how federal policies segregated Black Americans into increasing impoverished urban centers, resulting in generational cycles of concentrated poverty.^14^ Massey's empirical data showed that race and neighbourhood were so collinear as to make it ‘impossible to precisely estimate their separate effects’.^14^ More recent studies have found that multiple levels of neighbourhood segregation, including inter‐racial and intra‐racial income segregation, can be used to examine the more nuanced relationships among neighbourhood, race, and health outcomes, including obesity.^12^, ^13^, ^37^ While quantifying segregation is beyond the scope of this project, these neighbourhood differences, along lines associated with obesity risk, could explain why neighbourhood SEE functions efficiently as a summary measure for these demographic factors and can be used to identify concentrations of high risk for increased adiposity and obesity.

The finding that breastfeeding duration at least partially mediated the effects of SEE on obesity risk is important, as the downstream effects of increasing breastfeeding duration extend beyond obesity prevention. Increased breastfeeding duration is linked to a wide array of health benefits, including increased post‐partum weight loss and reduced risk of reproductive cancers for the mother and decreased risk of acute and chronic diseases and improved cognitive development in the infant.^38^ In addition, the implications of these findings extend beyond childhood, as early obesity is associated with increased risk of obesity and co‐morbid health conditions in adolescence and adulthood,^5^, ^7^ conditions that disproportionately affect populations of colour.^39^, ^40^ The significant differences in family‐level socio‐demographics by census tract, and the significant correlations with level of neighbourhood deprivation, provide a literal roadmap for identifying clusters of children at increased risk of obesity and other health disparities, while the finding that increased breastfeeding duration decreased risk of obesity in these neighbourhoods provides a method to at least partially address it. Thus, using the Deprivation Index to identify neighbourhoods for breastfeeding promotion and support may have consequential and long‐lasting benefits for the communities most at‐risk for a wide array of health disparities, including, but not limited to, a reduction in obesity rates.

Our analysis has some limitations. Our study focused on the predictive power of SEE regarding adiposity and obesity, as well as the influence of breastfeeding on obesity risk, ideally for use as a tool to identify loci and methods for health promotion. Thus, we did not test for interaction between SEE and the family‐level predictors, which may provide more insight into causality. Furthermore, physical environment and domestic stressors, such as neighbourhood crime, financial hardship and food security have been significantly associated with increased risk of obesity in older children.^9^, ^41^, ^42^ We did not include any measures of physical structural environment, crime, or food security in our models. However, given the significant correlations among individual family‐level predictors and neighbourhood SEE, it is probable that other measures of environmental and family stress would correlate with the Deprivation Index score and may be indirectly accounted for in our models. Further research is needed including these structural and environmental measures.

As a secondary analysis of PREVAIL, which aimed to characterize the natural history of respiratory and enteric viral illness in young children, potentially important variables for understanding differences in breastfeeding behaviours, such as timing of maternal return‐to‐work, availability of lactation support, or employment type, were not available and not included in any of the breastfeeding models. As this and other research shows that improving breastfeeding duration may reduce risk of obesity, studies to identify barriers to breastfeeding in these populations are needed to strategize best practices for improving these outcomes.

Finally, while PREVAIL's final enrollment was representative of the birth hospitals from which we recruited,^21^ our cohort was over‐represented by low‐income families compared to our region as a whole.^43^ This both widened the quartile ranges for Deprivation Index strata and drove the cut‐points for the categories down, resulting in more racial and economic diversity in the higher SEE quartiles than is representative of our area. Despite this possible misclassification of lower SEE neighbourhoods into our higher SEE categories, we found a strong relationship between SEE and child adiposity and obesity risk. Studies including a larger, more representative sample from a wider geographical area are needed to explore these relationships more fully.

## 5 CONCLUSIONS

Our approach showed that increased early childhood adiposity and obesity risk are associated with residing outside of the most affluent neighbourhoods as measured by level of material neighbourhood deprivation. We identified shorter breastfeeding durations outside of the highest SEE neighbourhoods as a partial mediator for obesity disparities. Our findings suggest that focusing breastfeeding support and promotion in the highest deprivation neighbourhoods may be an important first step to mitigate early childhood obesity and reduce significant health disparities in at‐risk populations, both in childhood and later in life.

## CONFLICT OF INTEREST

The authors declare that they have no known competing financial interests or personal relationships that could have appeared to influence the work reported in this paper.
